# A Description of Novel Uses of Hip Protectors in an Elderly Hip Fracture Population: A Technical Report

**DOI:** 10.7759/cureus.21028

**Published:** 2022-01-08

**Authors:** Patrick Nolan, Lauren Tiedt, Prasad Ellanti, Tom McCarthy

**Affiliations:** 1 Trauma and Orthopaedics, St. James's Hospital, Dublin, IRL; 2 Orthopaedics, National Orthopaedic Hospital Cappagh, Dublin, IRL; 3 Orthopaedics, St. James's Hospital, Dublin, IRL

**Keywords:** surgical wound management, secondary prevention, falls risk, hip protector, hip fracture

## Abstract

Hip fractures are a significant cause of morbidity and mortality in the elderly population. The number of hip fractures is set to increase significantly by 2050 as the global population ages. The costs associated with hip fracture patients are significant due to prolonged hospitalisation and rehabilitation.

Hip protectors have been advocated as a strategy to reduce the risk of hip fractures in a high-risk population. Evidence suggests that hip protectors are a cost-effective method for reducing the risk of hip fractures. There have, however, been issues with adherence with wearing hip protectors amongst patients and healthcare staff.

Despite prevention strategies, many patients continue to present with hip fractures. Many of these patients have cognitive impairment or experience peri-operative delirium. This can cause issues with patients' interference with the operative wound and presents a significant burden to the healthcare team with the need for increased wound monitoring and care in the post-operative period. Applying a well-fitted hip protector provides a substantial additional barrier to protect the surgical wound. Hip fracture surgical wounds can be difficult to manage in these patients and our standard post-operative protocol is to apply compression dressings in this group of patients. We have found that a well-fitted hip protector can provide adequate compression to the surgical site.

We describe a brief technical report on a novel use of hip protectors in providing wound security in the agitated patient post-operatively as well as a method of providing compression to the surgical wound site.

## Introduction

Hip fractures are a significant cause of morbidity and associated mortality in the elderly population. The global number of hip fractures is expected to increase from 1.26 million in 1990 to 4.5 million by 2050 [1]. There is a significant financial cost associated with these patients due to prolonged hospitalisation and rehabilitation.

Hip protectors were thought to be a cost-effective way of preventing hip fractures in the elderly population. Hip protectors are specially designed underwear with either hard or soft pads intended to reduce the forces transmitted to the proximal femur following a fall. Studies suggest that the use of hip protectors reduce the risk of hip fractures in elderly patients residing in nursing home or residential care settings [2].

Despite primary prevention efforts in these patients, many patients continue to present to the hospital with hip fractures every year. Up to 25% of these patients will experience peri-operative delirium [3]. In a cohort of patients frequently presenting with multiple risk factors for wound complications including co-morbidities and anticoagulants, non-compliance with post-operative wound care can lead to problems that represent a significant challenge to the orthopaedic surgeon.

In addition to their intended function, we describe the novel use of hip protectors in protecting post-operative wounds in an elderly cohort with cognitive impairment.

## Technical report

Hip protectors are routinely used in our institution in post-operative patients who are at high risk of falls. The brand available in our institution is the SafeHip© protector (Tyex, Ikast, Denmark). These hip protectors utilise soft foam padding and a variety of sizes ranging from extra-small to extra-extra-large are available. These sizes can facilitate a size of 65 cm (26 inches) up to 150 cm (60 inches).

We utilise the hip protectors on patients who present to our institution with a hip fracture and who undergo surgery and have a pre-existing diagnosis of cognitive impairment or prolonged post-operative delirium whose agitated state makes wound management difficult. Lacking awareness and insight, some of these often distressed patients can remove and or inadvertently interfere with the surgical site. This can lead to higher instances of delayed wound healing, discharge, and, ultimately, surgical site infection. Furthermore, many of these patients are on anticoagulants and antiplatelet therapy making their wounds prone to persistent wound ooze early in the postoperative period and the need for compression at the wound site.

**Figure 1:**
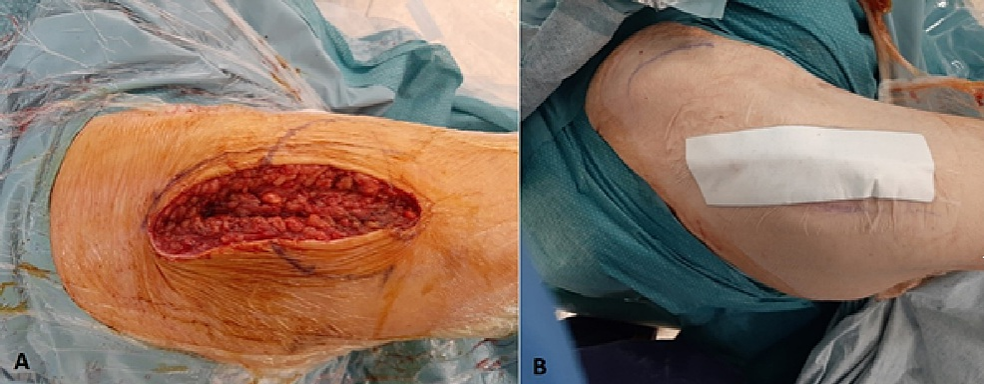
(A) Operative wound of a patient undergoing hemiarthroplasty. (B) The standard dressing used for wounds in hip fracture patients post-operatively.

Following the closure of the operative wound and application of a standard adhesive dressing (Figures 1A, 1B), the patient is placed into a hip protector of an appropriate size.

**Figure 2:**
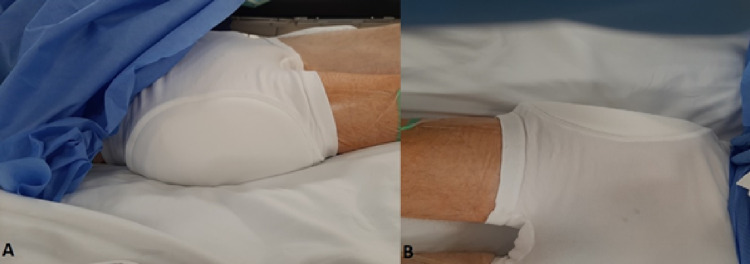
(A) and (B) Fitting of a standard hip protector in the operating room for a patient having undergone hip hemiarthroplasty.

To determine the correct size for the patient, a measurement should be taken at the widest point of the hips. The hip protector is applied on the operating table prior to transfer out of the theatre (Figures 2A, 2B).

Routine care of the operative wounds is easily performed in the post-operative setting as the hip protectors are readily manageable by the nursing staff.

The majority of patients for whom this novel use of hip protectors was implemented were post hemiarthroplasty; however, this also works well for those who have had a dynamic hip screw or for proximal wounds of those who have had a cephalomedullary nail. Despite significant issues with agitation, the author’s experience thus far is that patients have tolerated the hip protectors well and they have provided compression at the wound site and prevented any interference with the adhesive dressing or underlying wound.

Patients with urinary catheters may require a simple opening to be made at the front of the hip protector. While those who are incontinent can simply have their incontinence worn underneath the hip protector. These issues are easily manageable until the indication for the hip protector resolves, or the wounds heal.

## Discussion

Hip fractures in the elderly population usually occur following a fall resulting in a direct impact over the greater trochanter of the femur [4]. Hip protectors are medical devices that were designed to reduce the forces transmitted to the proximal femur following a fall and have been advocated as a strategy to reduce the risk of hip fractures in a high-risk elderly population. In 1993, Lauritzen et al. conducted the first clinical trial to report positive outcomes with the use of hip protectors in nursing home residents [5]. Korall et al. found that the wearing of a hip protector can reduce the risk of a hip fracture at the time of a fall by nearly three-fold [6].

There are many varieties of hip protectors available throughout the world. They can be broadly classified into hip protectors where padding with soft, energy-absorbing material is used and hip protectors where semi-rigid material is used that diverts the force away from the trochanteric region to the soft tissues of the thigh [7]. Keenan and Evans performed biomechanical testing using a standardised test to compare individual hip protectors in their effectiveness in preventing hip fractures [8]. They found that there were significant differences in the effectiveness of individual hip protectors in their ability to reduce the impact forces [8].

A systematic review undertaken by de Bot et al. concluded that the use of hip protectors in an elderly population at high risk for hip fractures, such as nursing home residents or patients in a geriatric ward in a hospital, was a cost-effective approach to preventing hip fractures [9]. Another study performed by Singh et al. found that the use of hip protectors was a more cost-effective method compared to no treatment or the use of calcium and vitamin D supplements [10].

When worn correctly, hip protectors have been found to be effective in reducing the risk of hip fractures in a high-risk population. One common issue with hip protectors, however, has been with adherence and acceptance from both patients and staff [11]. Strategies such as providing hip protectors at no cost to patients and providing educational sessions for patients and staff have been shown to improve adherence [12].

Patients at high risk for hip fractures include patients residing in long-term care facilities and geriatric wards in hospitals. Despite prevention efforts including hip protectors and osteoporosis treatment, patients will continue to present to the hospital with hip fractures. According to the National Hip Fracture Database, up to 25% of these patients will experience peri-operative delirium [3]. These patients will often have multiple co-morbidities and are often prescribed anticoagulants and other medications that are risk factors for poor wound healing. Post-operative wounds for these patients can be significant and are often only covered by a standard adhesive dressing as shown in Figures 1A, 1B. Agitation and delirium in combination with patient risk factors for poor wound healing can result in serious wound complications if the wound is not adequately managed. This can present a significant burden to nursing staff or healthcare assistants tasked with preventing any interference with the post-operative wound.

Surgical site infections (SSI) can have an enormous impact on patient outcomes and healthcare costs. Jenks et al. carried out a study in one English hospital and found that there were significant additional costs associated with SSI with a median additional cost of £4,656 for a superficial SSI and £6,690 for a deep or organ space SSI [13]. The average cost of using a hip protector is estimated in one study at £113 per person per year [14]. Hip protectors are a low-cost intervention that not only offers a cost-effective method for hip fracture prevention in high-risk patients but may also offer additional protection against SSI in this high-risk population and the significant costs associated with managing these complications. The authors have not yet experienced any complications resulting from the use of hip protectors.

We describe the novel use of hip protectors in patients with hip fractures post-operatively to protect the post-operative wound from patient interference due to cognitive impairment or delirium. Patients who have advanced cognitive impairment or who demonstrate peri-operative delirium are often non-compliant with post-operative wound care. The fitting of hip protectors in these patients presents a barrier to interference. Figures 2A, 2B demonstrate the fitting of the hip protector in a patient post-operatively. It demonstrates how the padding of the hip protector protects the wound from any interference as well as from any inadvertent impact that may occur. Additionally, the elastic material of the underwear provides some degree of compression, which may help with haemostasis. Of course, as well as this novel use of hip protectors, they may also be used for their intended purpose in this high-risk population to reduce the risk of hip fractures in the contralateral side and reduce the risk of injury to the operated side.

## Conclusions

In summary, management of post-operative wounds in an elderly hip fracture population with cognitive impairment can be difficult and presents a significant burden to the healthcare team. We describe the novel use of hip protectors in these patients as it can offer additional protection as well as compression to the post-operative wound in addition to providing the intended function of hip protectors in reducing the risk of hip fractures in a high-risk population. We feel that this use adds further value to the use of hip protectors and hope that this paper can help to encourage improved utilisation of hip protectors in this high-risk population.
